# GUN1, a Jack-Of-All-Trades in Chloroplast Protein Homeostasis and Signaling

**DOI:** 10.3389/fpls.2016.01427

**Published:** 2016-09-22

**Authors:** Monica Colombo, Luca Tadini, Carlotta Peracchio, Roberto Ferrari, Paolo Pesaresi

**Affiliations:** ^1^Centro Ricerca e Innovazione, Fondazione Edmund MachSan Michele all'Adige, Italy; ^2^Dipartimento di Bioscienze, Università degli Studi di MilanoMilan, Italy

**Keywords:** nucleoid, GUN1, protein homeostasis, retrograde signaling, biogenic control

## Abstract

The *GENOMES UNCOUPLED 1 (GUN1)* gene has been reported to encode a chloroplast-localized pentatricopeptide-repeat protein, which acts to integrate multiple indicators of plastid developmental stage and altered plastid function, as part of chloroplast-to-nucleus retrograde communication. However, the molecular mechanisms underlying signal integration by GUN1 have remained elusive, up until the recent identification of a set of GUN1-interacting proteins, by co-immunoprecipitation and mass-spectrometric analyses, as well as protein–protein interaction assays. Here, we review the molecular functions of the different GUN1 partners and propose a major role for GUN1 as coordinator of chloroplast translation, protein import, and protein degradation. This regulatory role is implemented through proteins that, in most cases, are part of multimeric protein complexes and whose precise functions vary depending on their association states. Within this framework, GUN1 may act as a platform to promote specific functions by bringing the interacting enzymes into close proximity with their substrates, or may inhibit processes by sequestering particular pools of specific interactors. Furthermore, the interactions of GUN1 with enzymes of the tetrapyrrole biosynthesis (TPB) pathway support the involvement of tetrapyrroles as signaling molecules in retrograde communication.

## Introduction

Upon illumination, proplastids differentiate into functional chloroplasts in developing photosynthetic tissues of cotyledons, leaves, and stems (Jarvis and López-Juez, 2013). Chloroplast biogenesis also occurs during the growth of young green tissues, as cells expand and mature chloroplasts undergo division by binary fission (Okazaki et al., 2010). This process is characterized macroscopically by rapid greening of the young chloroplast and microscopically by the concomitant formation of thylakoid membranes and the reorganization of nucleoids, i.e., DNA-containing structures without defined boundaries, which differ in number, size, and distribution within plastids at different developmental stages, and harbor the plastid gene expression (PGE) machinery (Pfalz and Pfannschmidt, 2013; Melonek et al., 2016).

At the molecular level, this rather complex biogenic transition is achieved by cytosolic synthesis of chloroplast-targeted proteins, followed by import, assembly, folding, and degradation of unfolded/misfolded proteins (Jarvis and López-Juez, 2013). Indeed, the plastid genome itself (the plastome) comprises fewer than 100 protein-coding genes, and the vast majority of the 2000–3000 proteins that make up the chloroplast proteome are encoded in the nucleus (Richly and Leister, 2004). In particular, precursor proteins carrying N-terminal transit peptides initially interact with two multiprotein complexes termed Translocon at the outer envelope membrane of chloroplasts (TOC) and Translocon at the inner envelope membrane of chloroplasts (TIC), which facilitate their active transport through the chloroplast envelope, powered by an ATP import motor, consisting of the stromal heat-shock protein 93 (Hsp93), heat-shock protein 70 (Hsp70), and heat-shock protein 90 (Hsp90; Flores-Perez and Jarvis, 2013; Inoue et al., 2013; Shi and Theg, 2013a,b). Upon translocation, proteins are exposed to different proteolytic systems of prokaryotic origin, which are responsible for protein maturation, control of protein abundance, and removal of either misfolded or damaged components. Among these, the stromal protease Clp is a multimeric complex made of chaperones and serine protease subunits, which serve general housekeeping functions. In contrast, the thylakoid-associated FtsH (Filamentous temperature sensitive H) proteases are zinc-containing metalloendopeptidases that have both chaperone and proteolytic functions, and participate in the Photosystem II repair cycle, together with the DEG serine proteases (Kato and Sakamoto, 2010; Van Wijk, 2015).

Besides translation and post-translational processes, chloroplast biogenesis also requires transcriptional coordination of thousands of nuclear genes with the expression of the comparatively few plastid genes in order to meet the needs of the developing chloroplast (Chan et al., 2016; Kleine and Leister, 2016). This is achieved through extensive exchange of information between plastids and the nucleus, for instance, via biogenic retrograde signaling—a system in which developmentally relevant stimuli in plastids induce the accumulation of specific signaling molecules that relay information to the nucleus, and in turn adjust the expression of nuclear genes to the needs of the plastids (Pogson et al., 2008; Woodson and Chory, 2008; Chan et al., 2016).

During the last 30 years, experiments with the carotenoid biosynthesis inhibitor norfluorazon (NF) and the inhibitor of plastid translation lincomycin (LIN), each of which arrests chloroplast development at the proplastid stage and represses the expression of photosynthesis-associated nuclear genes (PhANGs), have provided insights into the plastid's biogenic retrograde pathways (Oelmüller and Mohr, 1986; Oelmüller et al., 1986).

Six *genome uncoupled* (*gun*) mutants have been characterized in *Arabidopsis thaliana* that fail to repress transcription of the nuclear gene *Lhcb1.2* after NF treatment, and are thus impaired in retrograde signaling (Susek et al., 1993; Mochizuki et al., 2001; Larkin et al., 2003; Koussevitzky et al., 2007; Adhikari et al., 2011; Woodson et al., 2011). Five of these genes, *GUN2-6*, were found to be involved in tetrapyrrole biosynthesis (TPB), whereas *GUN1*, which encodes a nucleoid-localized pentatricopeptide repeat protein (PPR), has been shown to have a role in PGE, and to act as an integrator of multiple retrograde signals, since *gun1* mutants are unique in exhibiting a *gun* phenotype in response to both norfluorazon and lincomycin (Gray et al., 2003; Koussevitzky et al., 2007). However, the exact molecular role of GUN1 remained enigmatic until the new insights provided by the recent identification of a set of GUN1-interacting proteins (Tadini et al., 2016; Table 1).

**Table 1:** **GUN1 interactors together with their functions and impacts on plant development**.

**Designation**	**AGI code**	**Mutant phenotype^a^**	**Molecular function/Defect**	**Nucleoid subunit^b^**	**Identification assay^c^**	**References**
**TRANSCRIPTION AND RNA METABOLISM**						
pTAC6/PAP8	AT1G21600	Albino	Low PEP activity	+	CoIP-MS	Pfalz et al., 2006; Steiner et al., 2011; Pfalz and Pfannschmidt, 2013
RH3/EMB1138	AT5G26742	Embryo lethal	RNA splicing of group II introns, assembly of the 50S ribosomal particle	+	CoIP-MS	Asakura et al., 2012; Majeran et al., 2012
AtPPR_3g49240/EMB1796	AT3G49240	Embryo lethal	n.d.	+	CoIP-MS	Cushing et al., 2005; Majeran et al., 2012
**TRANSLATION**						
rpl2	ATCG00830	n.d.	Promotes translation initiation	+	CoIP-MS	Manuell et al., 2007; Melonek et al., 2016
rps3	ATCG00800	Essential for cell survival in tobacco	Promotes translation initiation	+	CoIP-MS	Manuell et al., 2007; Fleischmann et al., 2011; Melonek et al., 2016
rps4	ATCG00380	Essential for cell survival in tobacco	Involved in the assembly of the 30S ribosomal particle; binds to16S rRNA	+	CoIP-MS	Rogalski et al., 2008; Shoji et al., 2011; Melonek et al., 2016
PRPL10/EMB3136	AT5G13510	Embryo lethal	Part of the L12 stalk and required for translation, since it recruits auxiliary translation factors such as cpIF2	−	CoIP-MS	Baba et al., 2006; Bryant et al., 2011; Shoji et al., 2011; Pfalz and Pfannschmidt, 2013
PRPS1	AT5G30510	n.d.	Promotes translation initiation	−	Y2H; BiFC	Manuell et al., 2007; Shoji et al., 2011; Tadini et al., 2016
cpIF2/FUG1	AT1G17220	Embryo lethal	Promotes translation initiation; leaky mutant alleles suppress leaf variegation in *var* mutants	−	CoIP-MS	Miura et al., 2007
**PROTEIN IMPORT, PROTEIN FOLDING, AND PROTEIN UNFOLDING/DEGRADATION**						
Hsp93-III/ClpC2	AT3G48870	Single mutant identical to WT; *hsp93-III hsp93-V* double mutant is embryo lethal	Cooperates with Tic110 and Tic40 in chloroplast protein import; chaperone in the Clp protease complex	−	CoIP-MS	Inaba et al., 2003; Kovacheva et al., 2005; Chou et al., 2006; Sakamoto, 2006; Kovacheva et al., 2007; Van Wijk, 2015
Hsp93-V/ClpC1	At5g50920	Single mutant exhibits a chlorotic phenotype; *hsp93-III hsp93-V* double mutant is embryo lethal	Cooperates with Tic110 and Tic40 in chloroplast protein import; chaperone in the Clp protease complex	+	CoIP-MS	Inaba et al., 2003; Kovacheva et al., 2005; Chou et al., 2006; Sakamoto, 2006; Kovacheva et al., 2007; Van Wijk, 2015; Melonek et al., 2016
Hsp70-1	AT4G24280	Single mutant exhibits variegated cotyledons, malformed leaves, growth retardation and impaired root growth; *hsp70-1 hsp70-2* double mutant is lethal	Involved in chloroplast protein import, folding and onward guidance of newly imported polypeptide chains	+	CoIP-MS	Su and Li, 2008; Shi and Theg, 2010; Su and Li, 2010; Liu et al., 2014; Melonek et al., 2016
Hsp70-2	AT5G49910	Single mutant identical to WT; *hsp70-1 hsp70-2* double mutant is lethal	Involved in chloroplast protein import, folding and onward guidance of newly imported polypeptide chains	−	CoIP-MS	Su and Li, 2008; Shi and Theg, 2010; Liu et al., 2014; Su and Li, 2010
ptCpn60α1	AT2G28000	Albino	Involved in folding and onward guidance of newly imported polypeptide chains; essential for plastid division in *A. thaliana*; involved in Rubisco and NdhH assembly	+	CoIP-MS	Gutteridge and Gatenby, 1995; Apuya et al., 2001; Suzuki et al., 2009; Peng et al., 2011; Flores-Perez and Jarvis, 2013; Melonek et al., 2016
ptCpn60β1	AT1G55490	Leaves of the *len1* mutant have wrinkled and irregular surfaces and display lesions due to spontaneous cell death	Involved in folding and onward guidance of newly imported polypeptide chains; essential for plastid division in *A. thaliana*; involved in Rubisco and NdhH assembly	−	CoIP-MS	Gutteridge and Gatenby, 1995; Boston et al., 1996; Kessler and Blobel, 1996; Jackson-Constan et al., 2001; Ishikawa et al., 2003; Ishikawa, 2005; Suzuki et al., 2009; Flores-Perez and Jarvis, 2013
**TPB ENZYMES**						
CHLD	AT1G08520	Albino	Encodes the D subunit of the Mg-chelatase enzyme, involved in chlorophyll biosynthesis	−	Y2H; BiFC	Strand et al., 2003; Tanaka et al., 2011
PBGD	AT5G08280	n.d.	Porphobilinogen deaminase activity. Enzyme in the tetrapyrrole biosynthesis pathway	−	Y2H; BiFC	Tanaka et al., 2011
UROD2	AT2G40490	n.d.	Uroporphyrinogen decarboxylase activity; Enzyme in the tetrapyrrole biosynthesis pathway	−	Y2H; BiFC	Tanaka et al., 2011
FC1	AT5G26030	No visible phenotype; overexpression of the *FC1* gene is responsible for the *gun6* phenotype	Encodes ferrochelatase I, involved in heme biosynthesis	−	Y2H; BiFC	Tanaka et al., 2011; Woodson et al., 2011
**DIVERSE FUNCTIONS**						
rbcL	ATCG00490	Essential for photoautotrophy	Large subunit of Rubisco	+	CoIP-MS	Phinney and Thelen, 2005; Majeran et al., 2012; Huang et al., 2013
ATP-synthase β subunit	ATCG00480	Essential for photoautotrophy	Beta subunit of the thylakoid ATP synthase complex	+	CoIP-MS	Phinney and Thelen, 2005; Pfalz et al., 2006; Majeran et al., 2012; Melonek et al., 2012; Huang et al., 2013
RER4	AT5G12470	Mutant exhibits stunted growth, weak leaf reticulation and smaller mesophyll cells	Integral component of chloroplast outer and inner envelope membranes; possibly involved in retrograde signaling, supply of metabolites, control of ROS	−	CoIP-MS	Perez-Perez et al., 2013
2-Cys PrxA	AT3G11630	Mutant exhibits increased tolerance to photo-oxidative stress	Involved in peroxide detoxification in the chloroplast; functions as a redox sensor and chaperone; controls the conversion of Mg-protoporphyrin monomethyl ester into protochlorophyllide	−	CoIP-MS	Stenbaek et al., 2008; Rey et al., 2007; Pulido et al., 2010; König et al., 2013; Dietz, 2016

Note that proteins Q9SIP7 (AT2G31610) and Q42112 (AT3G09200) reported to be identified in coimmunoprecipitates of GUN1-GFP (Tadini et al., 2016) are not listed in this Table, since they have been described as subunits of cytosolic ribosomes. Furthermore, the protein Q9C5C2 (AT5G25980) has not been included, since it localizes to the tonoplast (Agee et al., 2010).

n.d., not determined.

a Phenotype of knock-out mutants is described.

b Protein already identified as part of chloroplast nucleoid by proteomic approaches.

c Assays used to identify the corresponding protein as a GUN1 interactor: coimmunoprecipitation followed by mass spectrometry (CoIP-MS), yeast two-hybrid (Y2H) analysis, and Bimolecular Fluorescence Complementation (BiFC).

Based on the functions of these partners, GUN1 appears to take part in multiple processes essential for chloroplast biogenesis and maintenance of the chloroplast proteome. GUN1-mediated control of plastid ribosomal protein S1 (PRPS1) accumulation, together with co-immunoprecipitation (CoIP) of proteins involved in different steps of plastid translation, support the involvement of GUN1 in the regulation of plastid protein synthesis. Furthermore, the presence of several chaperones in the CoIP mixture suggests a role for GUN1 in the coordination of chloroplast protein import and protein degradation.

Intriguingly, several GUN1 interactors appear to accumulate to higher levels upon induction of the unfolded protein response (UPR) in *Chlamydomonas reinhardtii* chloroplasts, which is triggered upon conditional repression of the catalytic subunit of Clp protease (ClpP1; Ramundo et al., 2013; Ramundo and Rochaix, 2014; Rochaix and Ramundo, 2015). This finding suggests the possible involvement of GUN1 in the UPR signaling pathway.

In this review, we describe the functional roles of the different GUN1 protein partners and propose some testable hypotheses that should clarify the molecular role of GUN1 in chloroplast biogenesis and chloroplast protein homeostasis.

## GUN1 is found in plastid nucleoids and interacts with the transcriptional machinery

GUN1 encodes a member of PPR-containing protein family, which has a small MutS-related (SMR) domain at the C-terminal end and a plastid targeting signal sequence at its N terminus. PPR motifs have been shown to mediate interactions with nucleic acids, and the SMR domain is found in proteins that act in DNA repair and recombination. However, *in vivo* RNA and DNA immunoprecipitation on chip (NIP-chip), as well as one-hybrid assays, have failed to detect any stable interaction of GUN1 with nucleic acids (Tadini et al., 2016), in contrast to a previous report, in which a GUN1 fragment containing both the PPR and SMR domains was shown to bind DNA *in vitro* (Koussevitzky et al., 2007). Nevertheless, GUN1 appears to be associated with nucleoids in the chloroplast, and more specifically with the domain of active plastid transcription, as shown by the relatively large and distinct foci of a fluorescent GUN1-YFP (Yellow Fluorescence Protein) chimera that co-localize with a Plastid Transcriptionally Active Chromosome 2-Cyan Fluorescence Protein (pTAC2-CFP) fusion in chloroplasts of mesophyll cells (Koussevitzky et al., 2007). However, although the repertoire of nucleoid-associated proteins so far identified is quite extensive, the GUN1 protein is not listed in any of the chloroplast or nucleoid/pTAC proteomes published to date (for a review see Melonek et al., 2016), most probably because it accumulates in very small amounts at specific developmental stages or under particular physiological conditions. This inference is supported by CoIP experiments with a Green Fluorescence Protein (GUN1-GFP) fusion and subsequent mass spectrometry (MS), which identified several nucleoid subunits as interactors with GUN1 (Tadini et al., 2016; Table 1).

**Figure 1:**
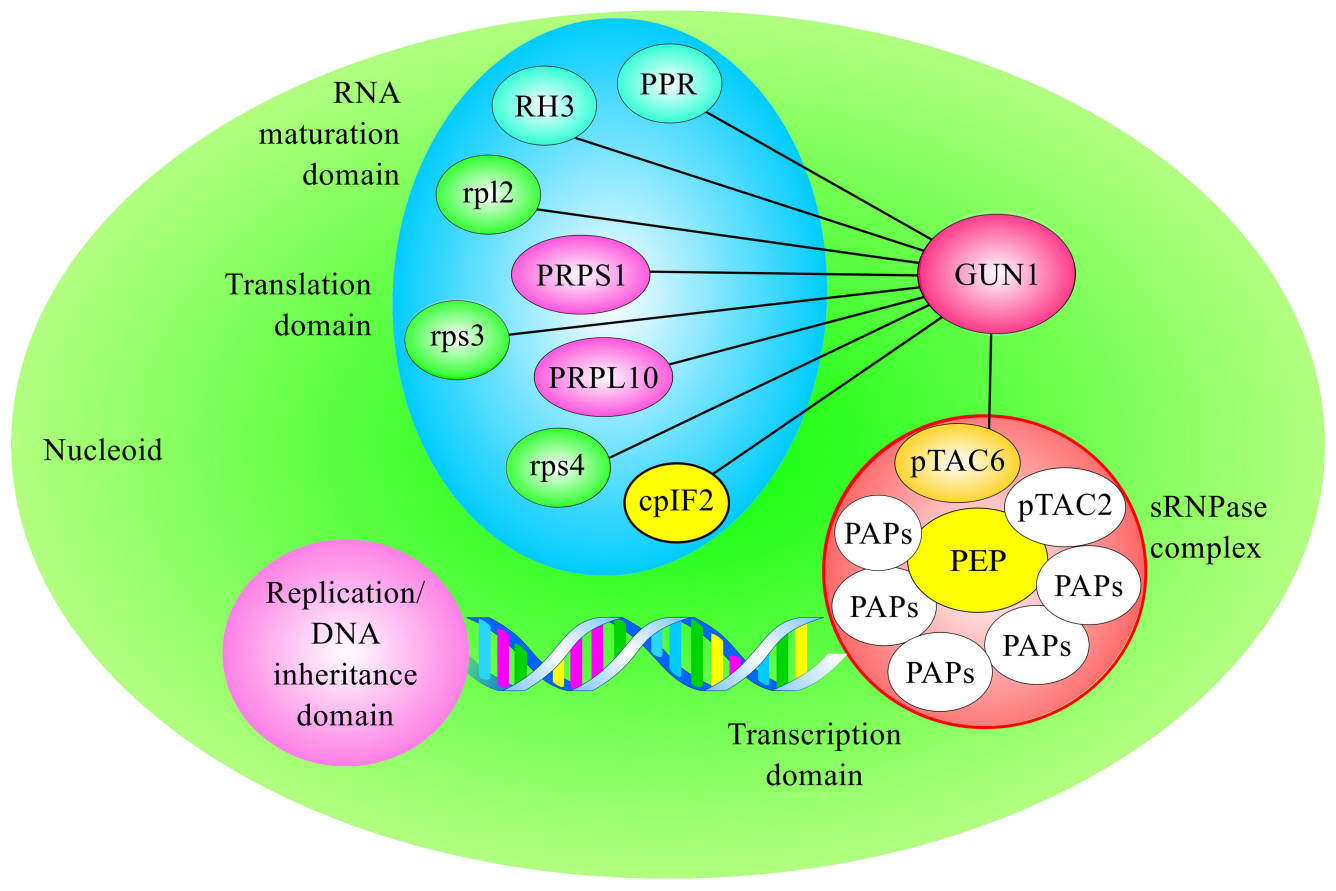
**Schematic overview of GUN1 protein interactors involved in gene transcription, ribosome biogenesis and plastid translation**. The scheme takes into account the partition of nucleoids into functional subdomains proposed by Pfalz and Pfannschmidt (2013). PPR refers to AtPPR_3g49240, also known as EMB1796, as reported in Table 1.

pTAC6 is among the GUN1 interactors, and it has been reported to interact directly with the plastid-encoded RNA polymerase (PEP), building together with pTAC2 and other polymerase-associated proteins (PAPs) the soluble RNA polymerase (sRNPase) complex (Pfalz et al., 2006), a central component of nucleoids (Steiner et al., 2011; Figure 1). Intriguingly, pTAC6 (also known as PAP8) contains no known domain and exhibits no homologies that could provide hints as to its function in PGE (Steiner et al., 2011). However, functional genomics analyses have indicated that homozygous *pap* knockout lines develop white cotyledons, fail to accumulate chlorophyll even under low light intensities, and do not produce primary leaves unless they are cultivated on MS medium supplemented with sucrose (for a review, see Pfalz and Pfannschmidt, 2013). Furthermore, analyses of PGE in *pap* mutants revealed strong repression of the accumulation of PEP-dependent transcripts, whereas levels of nucleus-encoded RNA polymerase (NEP)-dependent transcripts were not depleted, while some were enhanced, indicating that pTAC6/PAP8 and the other PAP proteins are essential for the activity of PEP (see Table 1).

## GUN1 controls plastid translation and ribosome biogenesis

GUN1 also interacts with several ribosomal subunits, such as the plastid-encoded ribosomal proteins L2, S3, and S4 (rpl2, rps3, and rps4) and the nucleus-encoded plastid ribosomal protein L10 (PRPL10; Figure 1). Furthermore, yeast two-hybrid and Bimolecular Fluorescence Complementation (BiFC) assays revealed a physical interaction between GUN1 and PRPS1 (Tadini et al., 2016). Ribosomal proteins have been reproducibly detected in nucleoid and pTAC proteomes (Melonek et al., 2016), further supporting the existence of a translational subdomain within the nucleoids, as proposed by Pfalz and Pfannschmidt (2013). The homologs of PRPL10, rpl2, PRPS1, rps3, and rps4 are essential components of the protein biosynthetic machinery in *Escherichia coli* (Baba et al., 2006; Shoji et al., 2011) and the indispensability of rps3 and rps4 has been also proven in tobacco plastids (Rogalski et al., 2008; Fleischmann et al., 2011). Furthermore, PRPL10 is annotated as EMBryo defective 3136 (EMB3136) in the SeedGenes Project database (http://www.seedgenes.org/), and in its absence Arabidopsis embryo development arrests at the globular stage (Bryant et al., 2011). Mutants devoid of PRPS1 have not been described. However, given the conservation of PRPS1 function in prokaryotes and chloroplasts, it can be confidently assumed that complete lack of PRPS1 is lethal in Arabidopsis.

Taking into consideration the function of these ribosomal proteins, it can be argued that their interaction with GUN1 has a dual purpose. On the one hand, GUN1 modulates protein synthesis by controlling the abundance of PRPS1, which, together with rps3 and rps2, has been reported to form the domain responsible for the interaction of the 30S ribosomal subunit with mRNA, promoting translation initiation (Manuell et al., 2007; Tadini et al., 2016). This role is supported further by the stable interaction of GUN1 with the chloroplast translation initiation factor 2 (cpIF2; Tadini et al., 2016), also known as FUG1, and reported to be essential for chloroplast biogenesis (Miura et al., 2007).

On the other hand, GUN1 seems to be involved in the process of ribosome biogenesis too, since nucleoid-associated ribosomes are thought to be in various stages of assembly, with several rRNA maturation steps occurring in a co-transcriptional and assembly-assisted manner, as in prokaryotic systems (Bohne, 2014). For instance, the DEAD-box-containing, ATP-dependent RNA helicase 3 (RH3), which has been functionally linked to the chloroplast nucleoid (Majeran et al., 2012), is among the proteins that interact with GUN1 (Tadini et al., 2016; see also Figure 1 and Table 1). RH3 is directly involved in the splicing of group II introns in the *rpl2, trnA, trnI*, and *rps12* transcripts and could be coimmunoprecipitated with immature and mature 23S rRNA (Asakura et al., 2012). Furthermore, the PPR protein At3g49240 also known as AtPPR_3g49240, according to the PPR protein database (http://www.plantenergy.uwa.edu.au/applications/ppr/ppr.php), is also part of GUN1's interactors, and its maize ortholog, GRMZM2G074599_P01, has been identified in the chloroplast nucleoid (Majeran et al., 2012). The gene is annotated as embryo defective 1796 (EMB1796) in the SeedGenes database, since the complete lack of AtPPR_3g49240 leads to the arrest of embryonic development at the globular stage (Cushing et al., 2005), further supporting the essential role of GUN1 interactors in chloroplast biogenesis.

## GUN1 and the import of chloroplast proteins

**Figure 2:**
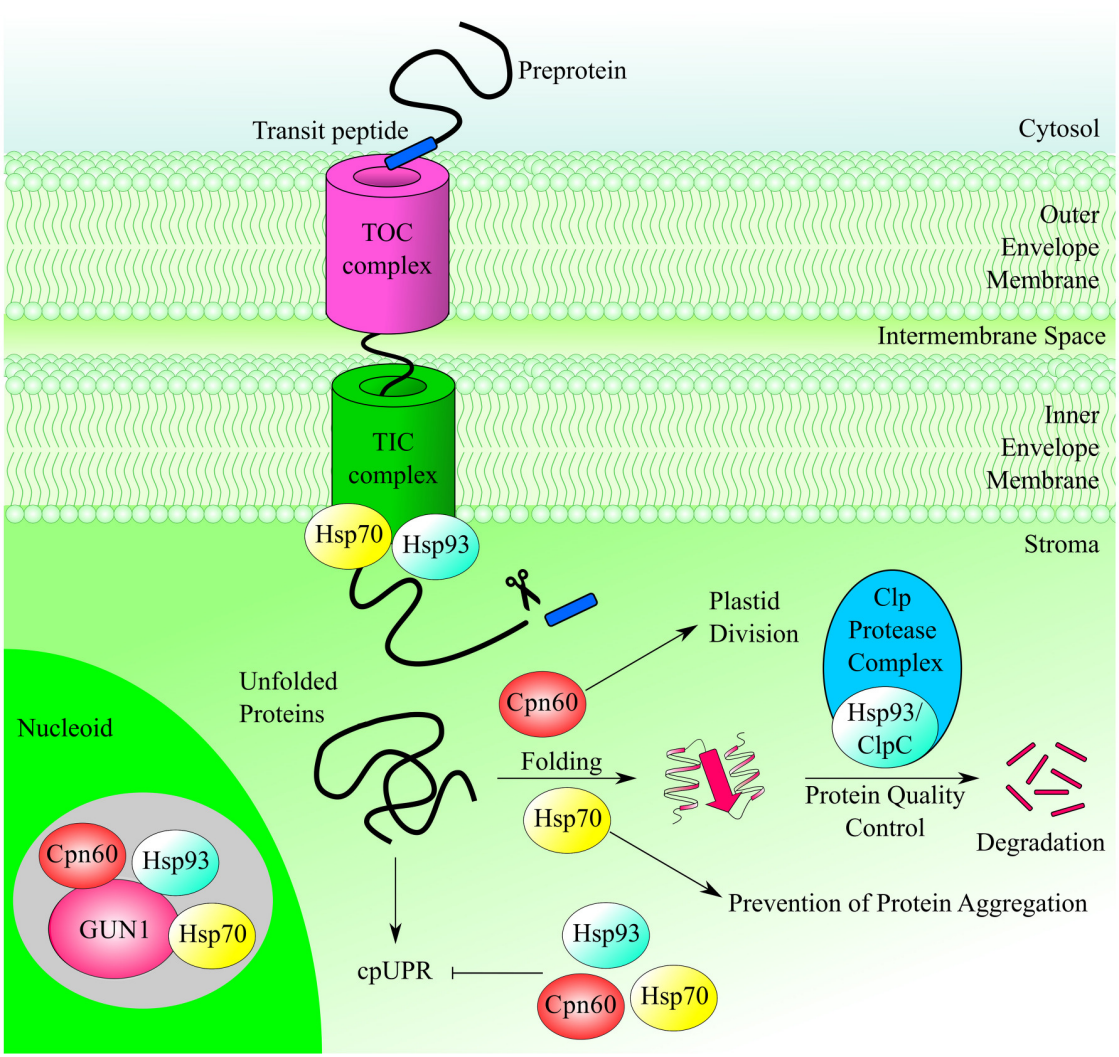
**GUN1 interacts with different plastid chaperones**. The chaperones Hsp93, Hsp70, and Cpn60 participate in different processes within the chloroplast, such as protein import, protein folding/unfolding, prevention of protein aggregation, and regulation of plastid division, and they might play a key role in the chloroplast Unfolded Protein Response (cpUPR). CoIP-MS analysis has shown that they are also part of GUN1-containing protein complexes.

Almost a quarter of the GUN1 interactors identified by CoIP-MS are chaperones (see Table 1), a relatively high proportion when compared with the extensive repertoire of protein functions found within the nucleoid (Melonek et al., 2016). The stromal Hsp93 and Hsp70 chaperones mediate different steps in protein import into the chloroplast stroma, whereas the 60 KD chaperonin Cpn60 is thought to be involved in the folding of newly imported mature proteins and to function downstream of Hsp93 and Hsp70 (Kessler and Blobel, 1996; Jackson-Constan et al., 2001; Flores-Perez and Jarvis, 2013). Furthermore, the two genes most highly co-regulated with *GUN1* encode the proteins TIC110 and TOC159 (Tadini et al., 2016), which are part of the outer and inner chloroplast translocons, respectively, suggesting a role of GUN1 in chloroplast protein import (Figure 2).

### The Hsp93 chaperones

In Arabidopsis, there are two nearly identical isoforms of Hsp93, termed Hsp93-V and Hsp93-III (or ClpC1 and ClpC2, respectively) and both interact with GUN1. The two proteins are highly homologous, but Hsp93-V is expressed at much higher levels than Hsp93-III (Kovacheva et al., 2005, 2007), and only Hsp93-V has been reported as a component of the nucleoid proteome (Phinney and Thelen, 2005; Majeran et al., 2012; Melonek et al., 2012; Huang et al., 2013). Furthermore, both *hsp93* single mutants are viable whereas *hsp93-III hsp93-V* double mutant is embryo-lethal, indicating that the two proteins have redundant functions in Arabidopsis chloroplasts (Constan et al., 2004; Sjögren et al., 2004; Kovacheva et al., 2007).

The current model for chloroplast protein import assumes that the preprotein transit peptide interacts with the TOC, and is subsequently transported through the TIC in an energy-dependent process (Shi and Theg, 2013b). In particular, the Tic110–Tic40 interaction is proposed to trigger the release of the transit peptide from Tic110 and enable the association of the preprotein with Hsp93 (Inaba et al., 2003). Tic40 then stimulates ATP hydrolysis by Hsp93, which harnesses the energy released to draw the preprotein into the stroma (Chou et al., 2006).

### The Hsp70 chaperones

Recent work has also demonstrated the involvement of Hsp70 in protein translocation into chloroplasts, as part of the translocon energy-dependent engine together with Hsp93 and Hsp90 (Inoue et al., 2013; Liu et al., 2014). Like Hsp93, Hsp70 proteins occur in two isoforms, Hsp70-1 and Hsp70-2, in the chloroplasts of Arabidopsis (Su and Li, 2008) and only Hsp70-1 was found in the proteomes of pTAC and crude nucleoids (for a review see Melonek et al., 2016). However, both Hsp70 proteins have been identified as GUN1 interactors (Tadini et al., 2016). Protein import assays using chloroplasts isolated from the Arabidopsis Hsp70 knockout mutants *hsp70-1* and *hsp70-2* showed that stromal Hsp70s are important for the import of both photosynthetic and non-photosynthetic precursor proteins, especially in early developmental stages (Su and Li, 2010). Furthermore, no *hsp70-1 hsp70-2* double mutant has ever been isolated. Thus, the two Hsp70s are likely to have redundant functions that are essential for plant development and chloroplast biogenesis.

### The Cpn60 chaperonins

After preproteins delivered to the stroma have been processed, they may require accessory factors to enable them to fold into their functional conformation, or to reach their final intra-organellar destination. The stromal molecular chaperones Hsp70, Cpn60, and Cpn10 are all believed to mediate the folding or onward guidance of newly imported polypeptide chains (Boston et al., 1996; Jackson-Constan et al., 2001). In particular, immunoprecipitation experiments have revealed that Cpn60 operates in close proximity with Tic110 (Kessler and Blobel, 1996), while import experiments have shown a transient association of mature, newly imported proteins with the Cpn60-Tic110 complex, suggesting that Tic110 can recruit Cpn60 in an ATP-dependent manner for the folding of proteins upon their arrival in the stroma. It has also been suggested that stromal Hsp70 and Cpn60 act sequentially to facilitate the maturation of imported proteins, particularly those destined for the thylakoid membranes (Madueno et al., 1993; Tsugeki and Nishimura, 1993; Peng et al., 2011). The Arabidopsis genome encodes two members of the Cpn60α family, denoted ptCpn60α1 and ptCpn60α2, and four members of Cpn60β, known as ptCpn60β1–β4 (Suzuki et al., 2009). Two of them, ptCpn60α1 and ptCpn60β2, have been linked to the nucleoid proteome (Melonek et al., 2016), and ptCpn60α1 and ptCpn60β1 are among the GUN1 interactors identified via the CoIP-MS strategy (see Table 1). The complete loss of ptCpn60α1, in the mutant termed *schlepperless* (*slp*), causes retardation of embryo development before the heart stage and an albino seedling phenotype, indicating that ptCpn60α1 is essential for chloroplast biogenesis (Apuya et al., 2001). Conversely, plants devoid of ptCpn60β1, also known as *lesion initiation 1* (*len1*), have leaves with wrinkled and irregular surfaces and undergo localized, spontaneous cell death in the absence of pathogen attack, i.e., lesion formation, under short-day conditions (Ishikawa et al., 2003).

## Other functions of plastid chaperones

Besides their roles in plastid protein import, all GUN1-interacting chaperones are present in the stroma at significant amounts relative to their association with the chloroplast import apparatus and perform various other functions together with different protein complexes (Figure 2). For instance Hsp93, also termed ClpC, acts as a regulatory chaperone in the Clp protease complex, the most abundant stromal protease with general household functions (Sakamoto, 2006; Van Wijk, 2015). Clp substrates are selected through various signals intrinsic to amino acid sequences and the ATP-dependent ClpC chaperone activity helps to progressively unfold selected substrates that are delivered to the ClpPR core for degradation into small peptides (~8–10 amino acids long; Olinares et al., 2011).

Similarly, Cpn60 forms a large oligomeric protein complex (>600 KDa) that promotes the assembly of Rubisco (Gutteridge and Gatenby, 1995). In particular, it has been observed that the large subunit of Rubisco (RbcL) is specifically associated with Cpn60 before assembly into the holoenzyme and that the Cpn60-RbcL complex is an obligatory intermediate. Furthermore, Cpn60 proteins have been shown to be essential for plastid division in *A. thaliana* (Suzuki et al., 2009). Thus, mesophyll cells in *ptcpn60*α*1-1* (a missense mutant) and *ptcpn60*β*1-1* (a protein null) plants, contain fewer and larger chloroplasts, indicating that normal levels of plastid Cpn60 are required for the correct folding of the stromal plastid division proteins and/or regulation of FtsZ (Filamentous temperature-sensitive Z) polymer dynamics (Suzuki et al., 2009).

The same holds true for the Hsp70 proteins, which are also involved in modulation of protein activity, regulation of protein degradation and prevention of irreversible protein aggregation when they are free in the stroma (Su and Li, 2008). Potentially GUN1 can be involved in a multitude of activities, besides plastid protein import, thus further investigations are needed to clarify the functional significance of GUN1–chaperone interactions.

## GUN1 and the chloroplast unfolded protein response (cpUPR)

Chaperones, together with enzymes that process and degrade proteins, are also necessary to maintain protein folding homeostasis in the various compartments of eukaryotic cells. Distinct signal transduction pathways, known as unfolded protein responses (UPRs), have evolved to couple the unfolded/misfolded protein load to the expression of specific chaperones and enzymes that promote folding and the disposal of misfolded proteins in each compartment.

The unfolded protein response was first discovered in the endoplasmic reticulum (ER) in yeast, where inhibition of protein folding leads to the transcriptional up-regulation of several chaperones (Cox et al., 1993), and subsequently in mitochondria, where accumulation of unfolded proteins in the mitochondrial matrix stimulates the expression of nuclear gene transcripts coding for mitochondrial chaperones (Aldridge et al., 2007; Lin and Haynes, 2016). Compared to yeast and metazoans, studies of plant UPRs are less advanced, and molecular details are known mainly for the ER-dependent UPR, which shows certain similarities with the process in multicellular eukaryotes, as well as plant-specific features (Ruberti et al., 2015). Recently, the possible existence of a chloroplast UPR (cpUPR) has been investigated in the green alga *Chlamydomonas reinhardtii*. Taking advantage of a repressible chloroplast gene expression system (Rochaix et al., 2014), Ramundo et al. (2014) induced the selective gradual depletion of the essential stromal Clp protease, in order to follow the early and late events caused by the decrease in its abundance. Temporal profiles of gene expression and protein accumulation revealed a marked increase in levels of chaperones, including Hsp70B, upon Clp depletion. Similar data have also been reported for Arabidopsis, where up-regulation of chloroplast chaperones and protein-sorting components occurred upon constitutive repression of Clp (Rudella et al., 2006; Zybailov et al., 2009). In particular, characterization of total leaf proteomes of WT and *clpr2-1* highlighted differential expression of 768 proteins. The largest functional category quantified (with 205 proteins) comprised proteins involved in translation, folding and degradation. Strikingly, all the chaperones interacting with GUN1, including Hsp93, Hsp70, Cpn60, as well as the DEAD box RNA helicase RH3, are among those up-regulated (by between 1.6- and 8.5-fold) in *clpr2-1* leaves, whereas no significant change in the chloroplast ribosomal protein population was observed (Zybailov et al., 2009).

Taken together, these findings suggest that disruption of protein homeostasis in organelles can be sensed and transduced to the nucleus to induce the expression of a specific set of factors responsible for promoting folding and monitoring protein quality control (Ramundo and Rochaix, 2014; Rochaix and Ramundo, 2015). After entering the higher plant chloroplast, these factors are able to interact with the nucleoid-associated GUN1 protein (Figure 2), which might therefore play a role in the cpUPR process.

## GUN1 and chloroplast metabolism

**Figure 3:**
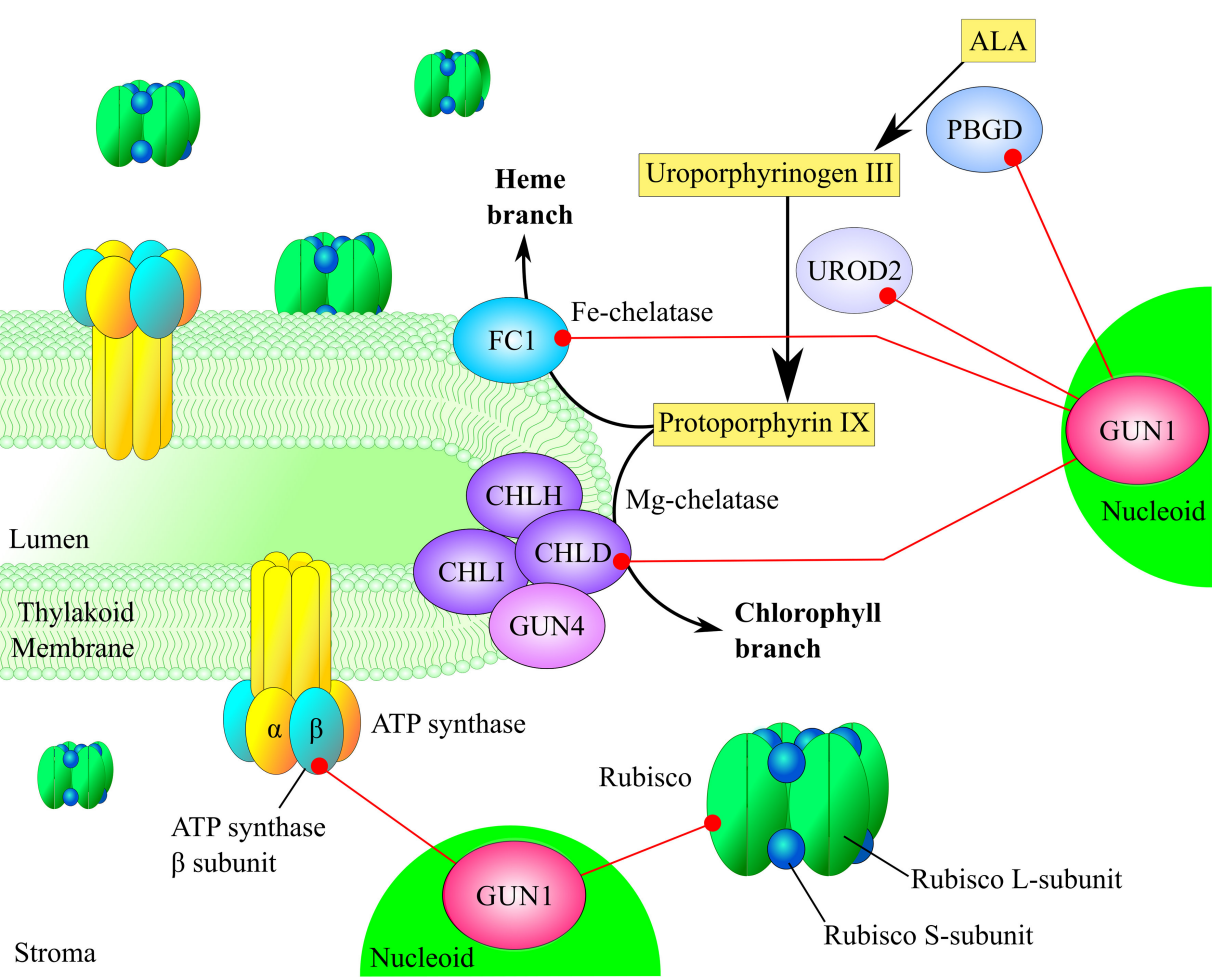
**GUN1 is involved in photosynthesis and tetrapyrrole biosynthesis**. The large subunit of Rubisco and the β-subunit of the thylakoid ATP synthase have been coimmunoprecipitated with GUN1, supporting a role for GUN1 in coordinating nucleoid activities with chloroplast metabolism. GUN1 also interacts with four enzymes of the tetrapyrrole biosynthesis pathway, i.e., the D subunit of Mg chelatase (CHLD), porphobilinogen deaminase (PBGD), uroporphyrinogen III decarboxylase (UROD2), and ferrochelatase I (FC1), as shown by yeast two-hybrid and Bimolecular Fluorescence Complementation. Note that the proteins RER4 and 2-Cys PrxA have not been included in this scheme for reasons of clarity.

The large subunit of ribulose bisphosphate carboxylase (RbcL) and the β subunit of the ATP synthase are also among the interactors of GUN1 identified by CoIP-MS analysis (Tadini et al., 2016). Because of their relatively high abundance in the chloroplast proteome, it is tempting to assume that these proteins are simply contaminants. However, RbcL and subunits of the ATP synthase have been repeatedly identified in the pTAC/nucleoid proteomes, even though different procedures were employed for isolation of crude nucleoid fractions and highly purified pTAC complexes (for a review see Melonek et al., 2016), thus suggesting these proteins might have a dual localization to the chloroplast stroma and nucleoids. The nucleoid association of RbcL and ATP synthase, i.e., of proteins that are not directly involved in core nucleoid functions, might also indicate that nucleoids also monitor photosynthesis and energy metabolism and respond appropriately to any perturbations (Figure 3).

Unlike RbcL and the ATP synthase β subunit, RETICULATA-RELATED 4 (RER4), an integral component of the chloroplast envelope membranes with three transmembrane α-helices, has never been identified in the pTAC/nucleoid proteome, although it appears to be an interactor of GUN1 (Table 1). The mutant *rer4-1* exhibits leaf reticulation, having green veins that stand out against paler intervein tissue, with fewer and smaller mesophyll cells than those of the wild type leaves (Perez-Perez et al., 2013). The molecular function of RER4 remains to be established. However, some hints as to its role in the chloroplast can be derived from features of the *rer4-1* mutant phenotype. A possible involvement of RER4 in retrograde signaling is suggested by the altered growth and development of mesophyll cells. Alternatively, the absence of RER4 might deplete the supply of essential metabolites during early stages of leaf development, which could explain the aberrant mesophyll structure. Furthermore, RER4 has been suggested to be involved in the control of reactive oxygen species (ROS), since the reticulated pigmentation of the *rer4-1* mutant grown under long-day conditions can be rescued by a short-day photoperiod, which markedly dampens ROS accumulation.

The 2-Cys peroxiredoxin A (2-Cys Prx A; see also Table 1), another interactor with GUN1, appears also to have a role in ROS scavenging (Rey et al., 2007; Pulido et al., 2010; Dietz, 2016) and, like RER4, it has never been reported to be part of the pTAC/nucleoid proteome (Pfalz et al., 2006; Majeran et al., 2012; Huang et al., 2013). 2-Cys Prx A and the highly homologous 2-Cys Prx B function as redox sensors and chaperones, thanks to the flexibility of their protein structure (König et al., 2013), and they have been shown to control the conversion of Mg-protoporphyrin monomethyl ester into protochlorophyllide (Stenbaek et al., 2008).

The involvement of GUN1 in TPB is further supported by its interaction with four TPB enzymes, namely subunit D of Mg chelatase (CHLD), porphobilinogen deaminase (PBGD), uroporphyrinogen III decarboxylase (UROD2), and ferrochelatase I (FC1), as demonstrated by both yeast two-hybrid and BiFC assays (Tadini et al., 2016; Figure 3). Interestingly, mutants defective in three of these GUN1 interactors—CHLD, PBGD, and FC1—have themselves been described as *gun* mutants (Strand et al., 2003; Huang and Li, 2009; Woodson et al., 2011), but have never been identified in crude nucleoid preparations, unlike subunit I of Mg chelatase (CHLI; Melonek et al., 2012; Huang et al., 2013).

## GUN1 and plastid protein homeostasis: some testable hypotheses

**Figure 4:**
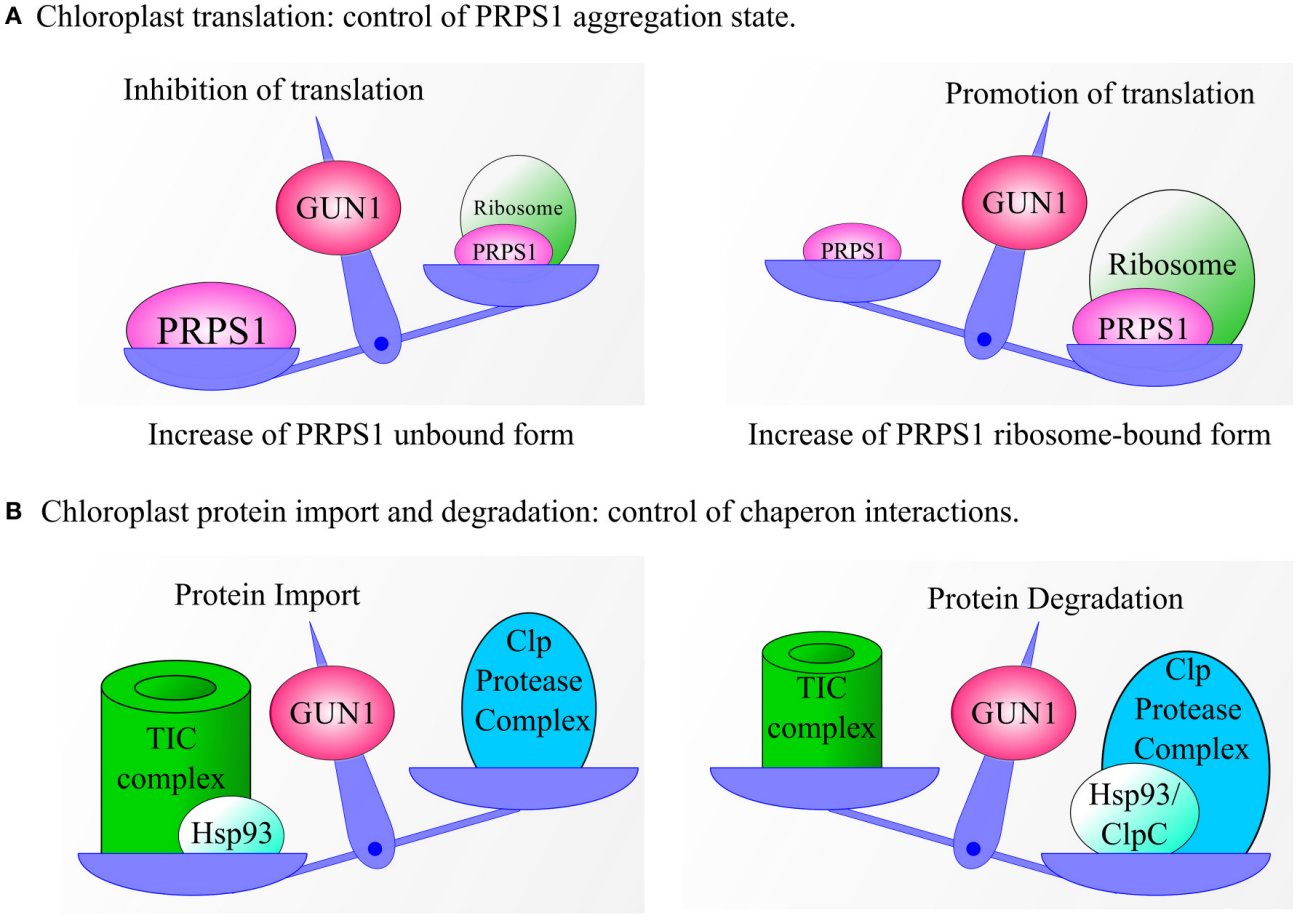
**Models explaining GUN1-dependent regulation of chloroplast translation, protein import and protein degradation. (A)** GUN1 controls the abundance of PRPS1 and its aggregation state. Increased levels of free PRPS1 prevent loading of mRNAs onto the ribosome and inhibit polysome formation, thus reducing overall rates of protein synthesis in the plastid. Conversely, when PRPS1 binds to ribosomes, polysome formation, and protein translation are stimulated. **(B)** Under certain conditions, the interaction between GUN1 and the Hsp93/ClpC protein might serve to bring the chaperone into close proximity with the TIC complex, thus favoring plastid protein import and reducing protein degradation. Alternatively, GUN1 could favor the interaction of Hsp93/ClpC with the Clp protease, thus promoting protein degradation at the expense of protein import. Note that a similar pattern of behavior can also be proposed for the other GUN1-interacting chaperones.

The recent identification of the GUN1 protein's partners in chloroplasts of Arabidopsis by means of CoIP-MS studies as well as in yeast two-hybrid and BiFC assays (Tadini et al., 2016) strongly suggests a major role for GUN1 in plastid protein homeostasis (Figure 4). This regulatory role involves proteins that are, in most cases, members of multimeric protein complexes and whose functions are often context-dependent. Furthermore, most GUN1 interactors appear to participate in four major processes:

### Chloroplast protein synthesis

A wealth of evidence accumulated during the last two decades supports the primacy of plastid protein synthesis in the control of chloroplast gene expression (Choquet and Wollman, 2002; Manuell et al., 2007; Tiller and Bock, 2014; Sun and Zerges, 2015). In this context, GUN1 has been suggested to regulate translation in plastids by modulating the abundance and binding affinity of PRPS1 (Tadini et al., 2016). In particular, PRPS1 is the only ribosomal protein that shuttles between ribosome-bound and ribosome-free forms (Merendino et al., 2003; Delvillani et al., 2011), the latter being more abundant in plants that lack GUN1. Based on observations in *E. coli*, where the unbound form is thought to inhibit translation by competing with ribosomes for mRNAs (Delvillani et al., 2011), it can be argued that the GUN1-dependent equilibrium between the two PRPS1 states has an important role in controlling polysome assembly and protein synthesis in chloroplasts (Figure 4A). However, further investigations are needed to clarify this issue. For instance, lines characterized by the ectopic expression of *PRPS1* or carrying *PRPS1* constructs under the control of inducible promoters, coupled with assays aimed to measure the translation rate in plastids, should allow us to verify the role of PRPS1 in modulating protein synthesis. Furthermore, GUN1 controls the abundance of PRPS1 at the post-transcriptional level. This suggests the involvement of an as yet unidentified plastid protease in this aspect of GUN1 function. In addition, the significance of the interaction of GUN1 with other ribosomal proteins, factors involved in ribosome biogenesis and regulators of plastid protein synthesis remains to be elucidated.

### Chloroplast protein import and degradation

Based on the observations reported above, it appears that GUN1 may well control the interactions of a sub-set of chaperones, promoting plastid protein import when their association with the TIC complex is favored, and stimulating protein degradation, folding/unfolding when they interact with proteases or other protein complexes in the stroma or in the thylakoid membranes (Figure 4B). Such a regulatory mechanism would enable GUN1 to coordinate protein translocation across the chloroplast envelope with protein degradation in the stroma, as well as with plastid division, thus modulating the protein content of the chloroplast in accordance with physiological requirements.

Relatively simple biochemical analyses can be used to verify the importance of GUN1 in influencing the interactions of the stromal chaperones, such as protein complex fractionation via sucrose-gradient ultracentrifugation and/or Blue-Native PAGE coupled with two-dimensional (2D) SDS-PAGE, and immunoblot analyses. Furthermore, the interactions of GUN1 with chaperones should be shown to occur at the plastid envelope and protein import efficiency should be tested in chloroplasts isolated from *gun1* and WT seedlings in order to implicate GUN1 in regulating plastid protein import.

### Retrograde signal induction

GUN1 may well be a master regulator of plastid-to-nucleus communication in *A. thaliana*, as it appears to integrate signals derived from perturbations in PGE, TPB, and redox state, in order to modulate nuclear gene expression. Indeed, components of all three pathways have been shown to interact with GUN1, suggesting that signal integration might take place through physical interaction.

Due to the limited abundance of GUN1, as indicated by the fact that the protein has yet to be detected in plastid proteome studies, it is tempting to disregard the idea that its physical interaction with PGE-, TPB-, and redox-related proteins could lead to protein sequestration and directly to differences in protein translation, TPB, and redox balance (Koussevitzky et al., 2007; Pogson et al., 2008; Woodson and Chory, 2008; Kleine and Leister, 2016). Nevertheless, a direct association with GUN1 could control protein abundance through post-transcriptional mechanisms, as in the case of PRPS1 and CHLD (Tadini et al., 2016). Thus, control of CHLD and possibly of FC1 levels could alter the tetrapyrrole flux and influence the abundance of the tetrapyrrole intermediate Mg-protoporphyrin IX (Mg-ProtoIX), or the tetrapyrrole product Fe-protoporphyrin IX (heme), which have been reported to act as negative and positive retrograde signals, respectively (for a review, see Chan et al., 2016). Alternatively, the interaction of GUN1 with the near-identical paralogs ClpC1 and ClpC2 could contribute to the coordination of plastid protein content with tetrapyrrole biosynthesis. Indeed, the activity of the stromal Clp protease has been shown to modulate tetrapyrrole flux by controlling (i) the accumulation of chlorophyll a oxygenase, which converts chlorophyll a into chlorophyll b (Nakagawara et al., 2007), and (ii) the level of glutamyl-tRNA reductase (GluTR), thus regulating the rate-limiting reaction in tetrapyrrole synthesis—the conversion of glutamate-1-semialdehyde into 5-aminolevulinic acid (Apitz et al., 2016).

Therefore, accurate determination of tetrapyrrole intermediates should be performed in *gun1* mutant and WT backgrounds. The analyses should be restricted to young seedlings or even to different developmental stages of the chloroplast, in line with the roles of tetrapyrrole and GUN1-mediated signaling in chloroplast development.

## Concluding remarks

In the past decade, substantial progress has been made in elucidating retrograde signaling, with the identification of multiple retrograde pathways and more than 40 components involved at different levels in chloroplast-to-nucleus communication. Nevertheless, the molecular function of GUN1 has remained unclear until the recent identification of the GUN1 protein's partners. Based on the functional roles of GUN1 interactors and the embryo lethal or albino phenotypes of most of the corresponding knock-out mutants, we have learned that GUN1 plays a role in chloroplast biogenesis, possibly by controlling protein turnover and protein import, and through the coordination of plastid and nuclear gene expression. Furthermore, GUN1 could have a role in the cpUPR process. Nonetheless, the involvement of GUN1 in plastid biogenesis and protein homeostasis is only just beginning to be understood. For instance, other approaches will be needed to validate the GUN1's protein partners identified by CoIP-MS. The use of a GUN1-GFP protein chimera, expressed under the control of a strong constitutive promoter such as the Cauliflower Mosaic Virus 35S (35S-CaMV), is indeed prone to the identification of false interactors. CoIP-MS studies using a GUN1 specific antibody appears to be the ideal strategy to identify protein partners. Alternatively, the use of GUN1 chimeras under the control of GUN1 native promoter is also practicable. Moreover, we do not know whether all these activities take place within one GUN1-containing nucleoid or if there are different nucleoids/locations for each GUN1-dependent function. The developmental stages of the chloroplast itself may even show distinct patterns of compartmentalization of the different functions. In addition, GUN1's interactions with its diverse partners might have quite different functional consequences: (i) promote specific functions, by bringing enzymes into close proximity with their own substrates and, ultimately, controlling the enzyme abundance, (ii) inhibit processes by sequestering sub-pools of specific proteins and, also in this case, controlling their abundance.

We are confident that future work, based on the exciting breakthroughs discussed in this Review, will shed new light on the molecular functions of GUN1 and its involvement in chloroplast biogenesis and protein homeostasis.

## Author contributions

MC, LT, CP, RF, and PP participated to the organization of the manuscript. MC and PP designed and conceived the pictures. PP wrote the manuscript.

### Conflict of interest statement

The authors declare that the research was conducted in the absence of any commercial or financial relationships that could be construed as a potential conflict of interest.
